# Diversity of *Monochaetia* Species from Fagaceous Leaf Spots in China and Pathogenicity for Chinese Chestnut

**DOI:** 10.1128/spectrum.00042-23

**Published:** 2023-04-04

**Authors:** Ning Jiang, Hermann Voglmayr, Han Xue, Chun-Gen Piao, Yong Li

**Affiliations:** a Key Laboratory of Biodiversity Conservation of National Forestry and Grassland Administration, Ecology and Nature Conservation Institute, Chinese Academy of Forestry, Beijing, China; b Department of Botany and Biodiversity Research, University of Vienna, Vienna, Austria; Agroscope

**Keywords:** molecular phylogeny, new species, plant disease, taxonomy

## Abstract

Pestalotioid fungi have been frequently studied with respect to their morphology, molecular phylogeny, and pathogenicity. *Monochaetia* is a pestalotioid genus that is morphologically characterized by 5-celled conidia with single apical and basal appendages. In the present study, fungal isolates were obtained from diseased leaves of *Fagaceae* hosts in China in 2016 to 2021 and identified based on morphology and phylogenetic analyses of the 5.8S nuclear ribosomal DNA gene with the two flanking internal transcribed spacer (ITS) regions, the nuclear ribosomal large subunit (LSU) region, the translation elongation factor 1-α (*tef1*) gene, and the β-tubulin (*tub2*) gene. As a result, five new species are proposed here, namely, Monochaetia hanzhongensis, Monochaetia lithocarpi, Monochaetia lithocarpicola, Monochaetia quercicola, and Monochaetia shaanxiensis. In addition, pathogenicity tests for these five species and Monochaetia castaneae from Castanea mollissima were conducted with detached leaves of Chinese chestnut. Results demonstrated that only M. castaneae successfully infected the host C. mollissima and caused brown lesions.

**IMPORTANCE** *Monochaetia* is a pestalotioid genus, with members that are commonly known as leaf pathogens or saprobes; some strains were isolated from air, in which case their natural substrate is so far unknown. *Fagaceae* represents an ecologically and economically important plant family that is widely distributed in the Northern Hemisphere, including an important tree crop species, Castanea mollissima, which is widely cultivated in China. In the present study, diseased leaves of *Fagaceae* in China were investigated, and five new *Monochaetia* species were introduced based on morphology and phylogeny of combined ITS, LSU, *tef1*, and *tub2* loci. Additionally, six species of *Monochaetia* were inoculated onto healthy leaves of the crop host Castanea mollissima to test their pathogenicity. The present study provides significant data on the species diversity, taxonomy, and host range of *Monochaetia* and enhances our understanding of leaf diseases of *Fagaceae* hosts.

## INTRODUCTION

The plant family *Fagaceae* is common and important, with 8 genera and about 927 species worldwide (1, 2). Many species in this family are economically beneficial to people, including, among others, the East Asian Chinese chestnut (Castanea mollissima), Quercus aliena, and Quercus variabilis. In China, C. mollissima is especially important for producing chestnut crops. Altogether, more than 320 members of *Fagaceae* are widely distributed in China.

Fungal species diversity occurring on fagaceous leaves has been recently studied based on morphological and phylogenetic features, revealing many interesting and new species of *Gnomoniopsis*, *Neopestalotiopsis*, *Pestalotiopsis*, *Tubakia*, and other genera (3–6). *Monochaetia* is a pestalotioid genus that was initially recognized as a subgenus of *Pestalotia* (as *Pestolozzia*), being characterized by the presence of a single apical appendage (7). Subsequently, *Monochaetia* was introduced as a distinct genus, including 23 species, but without indicating a type (8). Later, Monochaetia monochaeta was designated the type species of this genus (9). However, Steyaert (10) did not accept *Monochaetia* as a distinct genus and transferred its species into genera *Pestalotiopsis* and *Truncatella*. Contrariwise, Guba (11) reestablished *Monochaetia* for species having single apical and basal appendages. *Monochaetia* was restricted to species with 5-celled conidia, while species with 4-celled conidia were transferred to *Truncatella* or *Seimatosporium* and species with 6-celled conidia to *Seiridium* (12).

In recent molecular phylogenetic analyses of pestalotioid taxa, *Monochaetia* was confirmed as a separate genus of *Sporocadaceae* (13). Several new species were proposed based on morphology and phylogeny, for example, Monochaetia ilicis (as Monochaetia ilexae) from dead leaves of *Ilex* species, Monochaetia sinensis from dead leaves of *Quercus* species, Monochaetia castaneae from diseased leaves of Castanea mollissima, and Monochaetia schimae from diseased leaves of Schima superba (4, 14–16). The aims of the present study were to reveal hidden species diversity of *Monochaetia* from fagaceous leaves, to test their pathogenicity for the tree crop species Castanea mollissima, and to evaluate the practicability of host association for species distinction.

## RESULTS

### Phylogeny.

**FIG 1:**
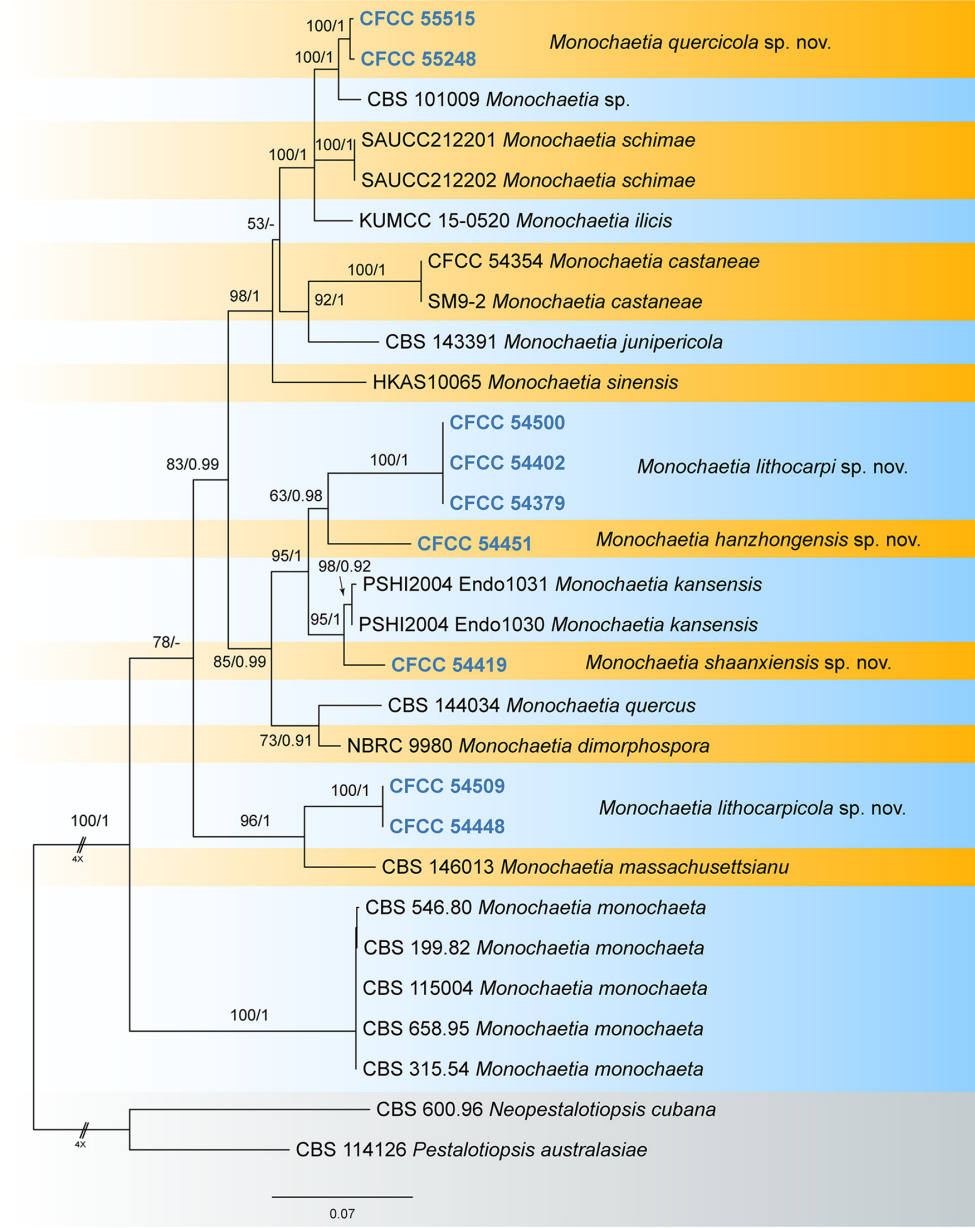
Phylogram of *Monochaetia* resulting from a ML analysis based on a combined matrix of ITS, LSU, *tef1*, and *tub2* loci. Numbers above the branches indicate ML bootstrap values of ≥50% (left) and BPP values of ≥0.9 (right). The tree is rooted with Neopestalotiopsis cubana (CBS 600.96) and Pestalotiopsis australasiae (CBS 114126). Isolates from the present study are marked in bold blue.

The combined sequence data set for the internal transcribed spacer (ITS), the large subunit (LSU), *tef1*, and *tub2* comprised 2,735 characters (563 for ITS, 838 for LSU, 523 for *tef1*, and 811 for *tub2*) from 29 isolates, including two outgroup taxa, namely, Neopestalotiopsis cubana (CBS 600.96) and Pestalotiopsis australasiae (CBS 114126). Of the 2,735 characters included in the phylogenetic analyses, 642 were parsimony informative (63 from the ITS, 34 from the LSU, 233 from *tef1*, and 312 from *tub2*). The best maximum likelihood (ML) tree (ln*L* = −11,824.18) revealed by RAxML is shown as a phylogram in Fig. 1. The topologies resulting from ML and Bayesian inference (BI) analyses of the concatenated data set were congruent. Isolates from the present study formed five individual clades representing five new species of *Monochaetia*, namely, Monochaetia hanzhongensis, Monochaetia lithocarpi, Monochaetia lithocarpicola, Monochaetia quercicola, and Monochaetia shaanxiensis.

### Pathogenicity.

**FIG 2:**
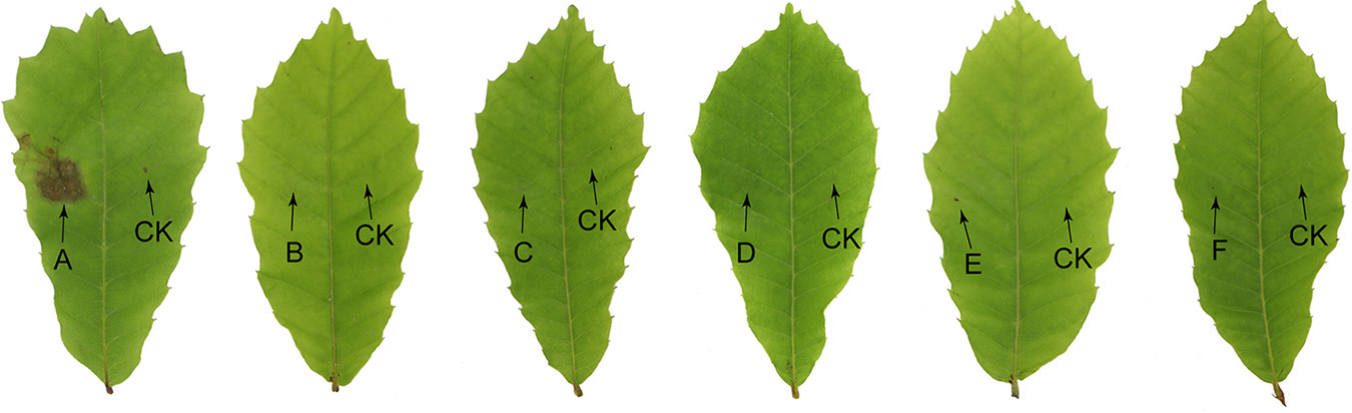
Results of pathogenicity tests with healthy leaves of Castanea mollissima after 10 days. (A) Monochaetia castaneae (CFCC 54354). (B) M. hanzhongensis (CFCC 54451). (C) M. lithocarpi (CFCC 54402). (D) M. lithocarpicola (CFCC 54509). (E) M. quercicola (CFCC 55515). (F) M. shaanxiensis (CFCC 54419). CK. negative control.

Ten days after inoculation, Monochaetia castaneae (CFCC 54354) produced brown lesions on the tested healthy leaves of Castanea mollissima. In contrast, there was no lesion development on the negative control or on leaves inoculated with M. hanzhongensis (CFCC 54451), M. lithocarpi (CFCC 54402), M. lithocarpicola (CFCC 54509), M. quercicola (CFCC 55515), or M. shaanxiensis (CFCC 54419) (Fig. 2). Reisolation was conducted using leaves inoculated with M. castaneae, and 5 isolates were obtained and identified as M. castaneae on the basis of the morphology of conidia and DNA sequence data. As a result, M. castaneae was confirmed as the leaf pathogen of C. mollissima, while the other five species of *Monochaetia* from the *Fagaceae* host failed to infect C. mollissima.

## TAXONOMY

**FIG 3:**
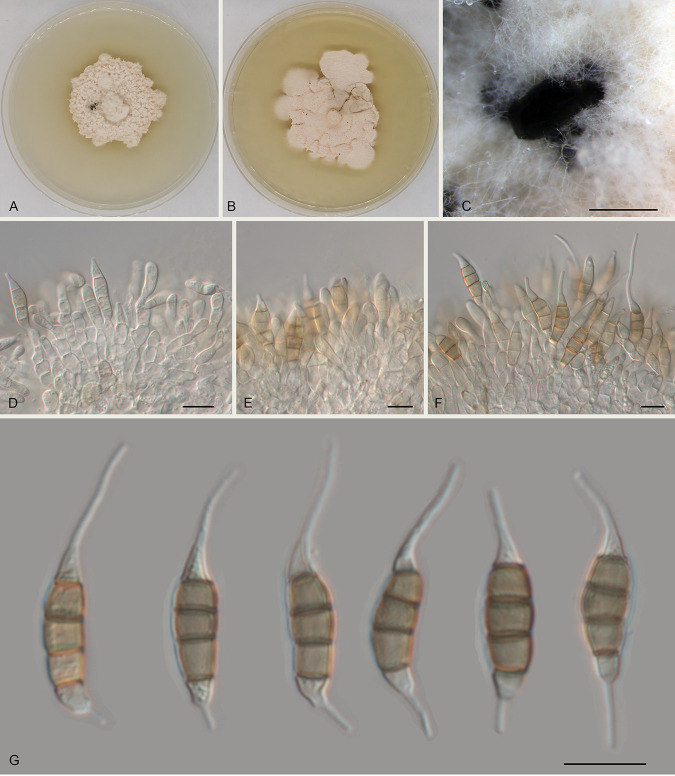
Morphology of Monochaetia hanzhongensis (CFCC 54451). (A) Colony on PDA after 10 days at 25°C. (B) Colony on MEA after 10 days at 25°C. (C) Conidioma formed on PDA. (D to F) Conidiogenous cells giving rise to conidia. (G) Conidia. Scale bars, 500 μm (C) and 10 μm (D to G).

Notes: Monochaetia hanzhongensis is phylogenetically close to Monochaetia dimorphospora, Monochaetia kansensis, M. lithocarpi, Monochaetia quercus, and M. shaanxiensis (Fig. 1). Morphologically, M. hanzhongensis is similar to M. lithocarpi in conidial size (17.5 to 21 by 5 to 6.5 μm versus 15 to 24.5 by 4.5 to 7 μm in M. lithocarpi) (Table 1) but can be distinguished from M. lithocarpi by sequence data (nucleotide differences in ITS: 10/524 nucleotides [1.91%] and a 32-bp insertion; *tef1*: 68/473 nucleotides [14.38%], 6-bp insertions, and 7-bp gaps; *tub2*: 39/421 nucleotides [9.26%] and 4-bp insertions). M. hanzhongensis differs from M. dimorphospora, M. kansensis, and M. shaanxiensis in conidial width (5 to 6.5 μm versus 4 to 4.5 μm in M. dimorphospora, 6 to 8 μm in M. kansensis, and 6.5 to 8.5 μm in M. shaanxiensis) and has shorter conidia than M. quercus (17.5 to 21 μm versus 22.5 to 29 μm in M. quercus) (Table 1) (17).

Monochaetia hanzhongensis Ning Jiang sp. nov. (Fig. 3). MycoBank number MB841304. Etymology: named after the collection site of the type specimen, Hanzhong City. Diagnosis: distinct from the phylogenetically related species of M. lithocarpi by host association. Typus: China, Shaanxi Province, Hanzhong City, Foping County, Dongshan Mountain, on diseased leaves of Quercus variabilis, 7 September 2019, Yong Li (holotype CAF 800016; ex-holotype culture CFCC 54451). Description: conidiomata in culture sporodochial, aggregated or solitary, erumpent, pulvinate, black, 300 to 850 μm in diameter, exuding black conidial masses. Conidiophores indistinct, usually reduced to conidiogenous cells. Conidiogenous cells hyaline, smooth, cylindrical to subcylindrical, annelidic, 5 to 13 by 3 to 4 μm, mean ± standard deviation (SD) = 10 ± 3 by 3.7 ± 0.3 μm. Conidia fusoid, straight or slightly curved, 4-septate, smooth, slightly constricted at the septa, without appendages (17.5 to) 18 to 20 (to 21) by (5 to) 5.5 to 6 (to 6.5) μm, mean ± SD = 19 ± 1.2 by 5.7 ± 0.3 μm (*n* = 50), length/width ratio (L/W) = 2.9 to 3.8; basal cell obconic with a truncate base, thin walled, hyaline or pale brown, 3 to 4.5 (to 5) μm long (without appendage); median cells 3, trapezoid or subcylindrical, brown, thick walled, the first median cell from the base (3.5 to) 4 to 5 (to 5.5) μm long, the second cell (3 to) 3.5 to 4 μm long, the third cell 3.5 to 4 μm long, altogether (10 to) 10.5 to 12.5 (to 13) μm long; apical cell conic with an acute apex, thin walled, hyaline, 2.5 to 4 μm long (without appendage); basal appendage single, unbranched, tubular, centric, straight or bent, (3 to) 3.5 to 9.5 (to 12) μm long, mean ± SD = 6.3 ± 3.4 μm; apical appendage single, unbranched, tubular, centric, straight or bent, (6.5 to) 7 to 18.5 (to 23) μm long, mean ± SD = 12.9 ± 5.7 μm. Sexual morph unknown. Culture characteristics: colonies on malt extract agar (MEA) flat, spreading, with flocculent aerial mycelium, folded surface, and diffuse edge, white to fawn, reaching 50 mm in diameter after 10 days at 25°C, forming black conidiomata with black conidial masses; colonies on potato dextrose agar (PDA) flat, spreading, with flocculent aerial mycelium and undulate margin, white to pale luteous, reaching 40 mm in diameter after 10 days at 25°C, forming black conidiomata with black conidial masses.

**TABLE 1:** Synopsis of *Monochaetia* species occurring on fagaceous hosts

Species	Host(s)	Distribution	Length of conidia (μm)	Width of conidia (μm)	Length of 3 median cells (μm)	Length of apical appendage (μm)	Length of basal appendage (μm)	Sequence data	Reference(s)
M. bicornis	*Quercus* sp.	Algeria	13–18	4–5	9–13	4–15	3–16	NA^*a*^	18
M. castaneae	Castanea mollissima	China	18.8–27.3	4.7–6.6	NA	17.5–35	10–20	ITS, LSU, *rpb2*, *tef1*, *tub2*	3
M. concentrica	*Castanea* sp., *Quercus* sp.	Alabama	20–26	6.5–8.5	15–19	15	NA	NA	18
M. hanzhongensis	Quercus variabilis	China	17.5–21	5–6.5	10–13	6.5–23	3–12	ITS, LSU, *tef1*, *tub2*	This study
M. hysteriiformis	*Quercus* sp.	USA	20–25	6.5–9.5	19–24	9–13	3–10	NA	11
M. kansensis	*Castanea* sp., *Quercus* sp.	USA	18–26	6–8	12–17	10–38	3–15	ITS, LSU, *tub2*	18
M. lithocarpi	Lithocarpus glaber	China	15–24.5	4.5–7	10–13	13–23.5	4–17	ITS, LSU, *tef1*, *tub2*	This study
M. lithocarpicola	Lithocarpus glaber	China	31–35	7.5–9	18–23	12–24	6.5–13.5	ITS, LSU, *tef1*, *tub2*	This study
M. monochaeta	*Quercus* sp.	France	15–21	5–8	10–15	5–19	NA	ITS, LSU, *rpb2*, *tef1*, *tub2*	8, 13
M. quercicola	Quercus acutissima, Q. aliena	China	19.5–25	7–9.5	13–15.5	5–17.5	5–11.5	ITS, LSU, *tef1*, *tub2*	This study
M. quercus	Quercus eduardi	Mexico	22.5–29	4.5–7	15–20	7–17.5	4.5–15	ITS, LSU, *rpb2*, *tef1*, *tub2*	13
M. shaanxiensis	Quercus baronii	China	19–22	6.5–8.5	11.5–13	3.5–8	2–3	ITS, LSU, *tef1*, *tub2*	This study
M. sinensis	*Quercus* sp.	China	25–31	8–10	18–19	17–32	10–20	ITS, LSU, *tub2*	15

a NA, not available.

**FIG 4:**
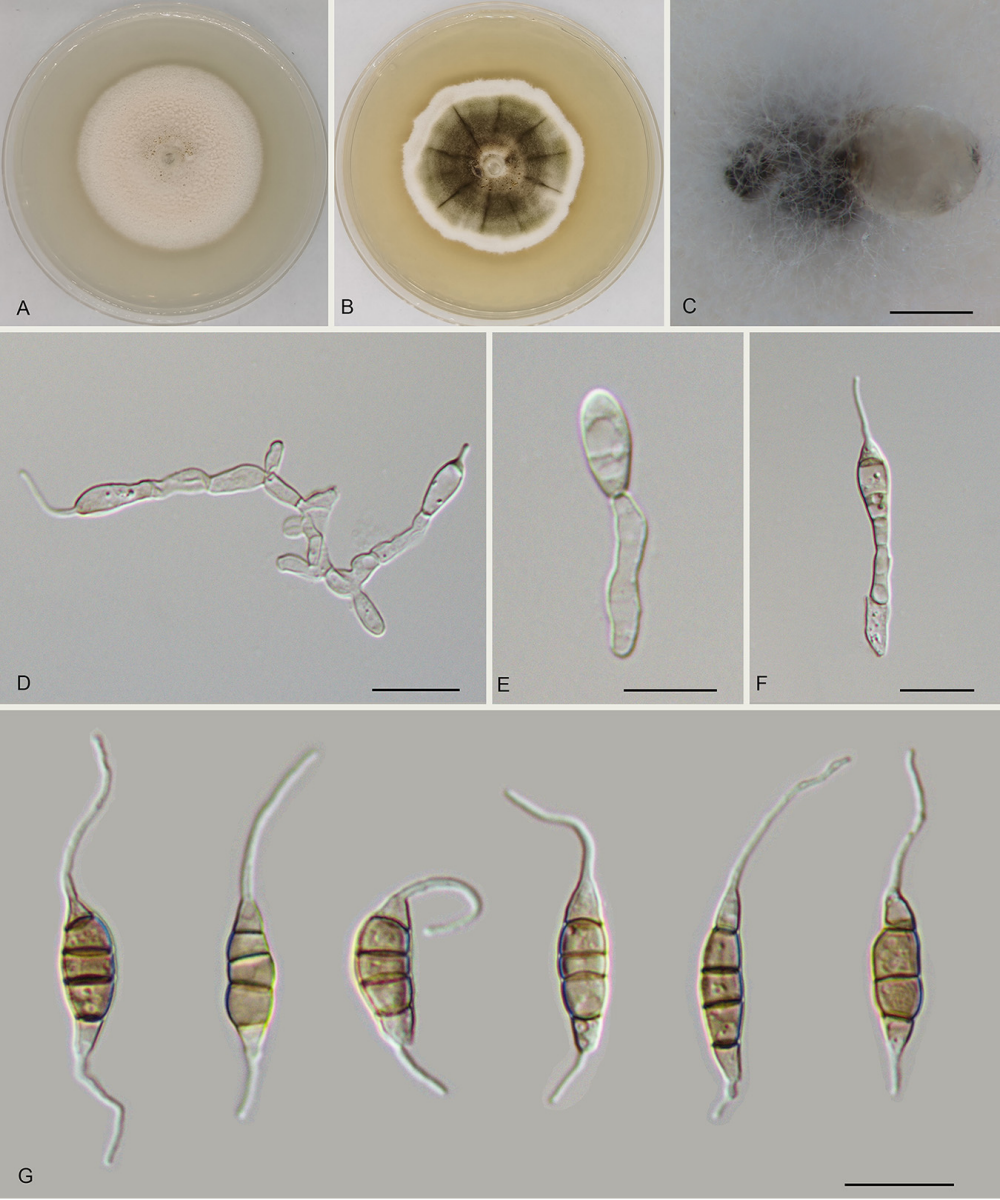
Morphology of Monochaetia lithocarpi (CFCC 54402). (A) Colony on PDA after 10 days at 25°C. (B) Colony on MEA after 10 days at 25°C. (C) Conidioma formed on PDA. (D to F) Conidiogenous cells giving rise to conidia. (G) Conidia. Scale bars, 300 μm (C) and 10 μm (D to G).

Notes: Three isolates from leaf spots of Lithocarpus glaber clustered into a distinct and well-supported clade in the phylogram, which is described here as the new species Monochaetia lithocarpi (Fig. 1). Monochaetia lithocarpi is phylogenetically close to M. dimorphospora, M. hanzhongensis, M. kansensis, M. quercus, and M. shaanxiensis (Fig. 1). Morphologically, M. lithocarpi has wider conidia than M. dimorphospora (4.5 to 7 μm versus 4 to 4.5 μm in M. dimorphospora) (18) and differs from M. quercus in the length of three median cells of conidia (10 to 13 μm versus 15 to 20 μm in M. quercus) (13). M. lithocarpi shares similar conidial size with M. hanzhongensis, M. kansensis, and M. shaanxiensis (17.5 to 21 by 5 to 6.5 μm in M. hanzhongensis versus 18 to 26 by 6 to 8 μm in M. kansensis versus 15 to 24.5 by 4.5 to 7 μm in M. lithocarpi versus 19 to 22 by 6.5 to 8.5 μm in M. shaanxiensis) (Table 1). However, M. lithocarpi can be distinguished by sequence data (nucleotide differences from M. hanzhongensis in ITS: 10/492 nucleotides [2.1%] and a 32-bp gap; *tef1*: 68/474 nucleotides [14.35%], 7-bp insertions, and 6-bp gaps; *tub2*: 39/417 nucleotides [9.35%] and 4-bp gaps; from M. kansensis in ITS: 5/483 nucleotides [1.04%], 1 insertion, and 34-bp gaps; *tub2*: 36 or 37/412 nucleotides [8.74 to 8.98%] and 5-bp gaps; from M. shaanxiensis in ITS: 12/492 nucleotides [2.44%], 1-bp insertion, and 33-bp gaps; *tef1*: 77/454 nucleotides [16.96%], 10 insertions, and 5 gaps; *tub2*: 38/417 nucleotides [9.11%] and 4-bp gaps).

Monochaetia lithocarpi Ning Jiang sp. nov. (Fig. 4). MycoBank number MB841305. Etymology: named after the host genus, *Lithocarpus*. Diagnosis: distinct from the phylogenetically related species of M. hanzhongensis by host association. Typus: China, Guangdong Province, Qingyuan City, Yangshan County, Guangdong Nanling Nature Reserve, on diseased leaves of Lithocarpus glaber, 4 December 2019, Shang Sun (holotype CAF 800017; ex-holotype culture CFCC 54402). Description: conidiomata in culture sporodochial, solitary, erumpent, pulvinate, dark brown, 250 to 600 μm in diameter, exuding dark brown conidial masses. Conidiophores septate and branched, hyaline or pale brown, thin walled. Conidiogenous cells hyaline or pale brown, smooth, cylindrical to subcylindrical, annelidic, 4.5 to 17 by 2 to 4 μm, mean ± SD = 10.3 ± 3.4 by 3 ± 0.6 μm. Conidia fusoid, straight or slightly curved, mostly 4-septate, occasionally 3-septate, wall smooth or undulate, not constricted at the septa but commonly collapsed at the septa, without appendages (15 to) 17 to 22 (to 24.5) by (4.5 to) 5 to 6.5 (to 7) μm, mean ± SD = 19.4 ± 2.6 by 5.8 ± 1.6 μm (*n* = 50), L/W = 2.6 to 4.6; basal cell obconic with a truncate base, thin walled, hyaline or pale brown, (2.5 to) 3 to 4.5 (to 5) μm long (without appendage); median cells 3 (rarely 2), trapezoid or subcylindrical, brown, thick walled, the first median cell from the base 4 to 5 μm long, the second cell (2.5 to) 3 to 4.5 (to 5) μm long, the third cell 3 to 4 μm long, altogether (10 to) 10.5 to 12.5 (to 13) μm long; apical cell conic with an acute apex, thin walled, hyaline or pale brown, 3 to 4 μm long (without appendage); basal appendage single, unbranched, tubular, centric, straight or bent, 4 to 13 (to 17) μm long, mean ± SD = 8.5 ± 3.3 μm; apical appendage single, unbranched, tubular, centric, variously bent, (13 to) 14.5 to 21.5 (to 23.5) μm long, mean ± SD = 17.8 ± 3.5 μm. Sexual morph unknown. Culture characteristics: colonies on MEA flat, spreading, with radially folded surface and undulate edge, olivaceous gray, reaching 35 mm in diameter after 10 days at 25°C, forming black conidiomata with black conidial masses; colonies on PDA flat, with moderate aerial mycelium and entire edge, white to pale luteous, reaching 40 mm in diameter after 10 days at 25°C, forming black conidiomata with black conidial masses. Additional materials examined: China, Guangdong Province, Qingyuan City, Yangshan County, Guangdong Nanling Nature Reserve, on diseased leaves of Lithocarpus glaber, 4 December 2019, Shang Sun (cultures CFCC 54379 and CFCC 54500).

**FIG 5:**
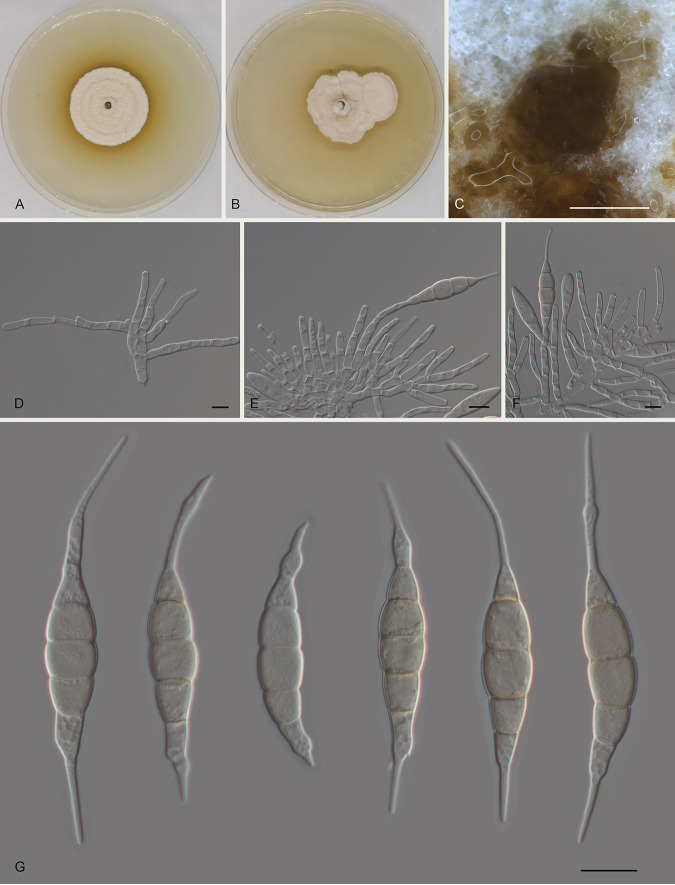
Morphology of Monochaetia lithocarpicola (CFCC 54509). (A) Colony on PDA after 10 days at 25°C. (B) Colony on MEA after 10 days at 25°C. (C) Conidioma formed on PDA. (D to F) Conidiogenous cells giving rise to conidia. (G) Conidia. Scale bars, 300 μm (C) and 10 μm (D to G).

Notes: In our phylogenetic analyses (Fig. 1), M. lithocarpicola is the closest relative of Monochaetia massachusettsianum, which was isolated from air in the United States and for which the natural host is therefore unknown. However, M. lithocarpicola is distinguished from M. massachusettsianum by obviously longer conidia (31 to 35 μm versus 23 to 30 μm in M. massachusettsianum) (19).

Monochaetia lithocarpicola Ning Jiang sp. nov. (Fig. 5). MycoBank number MB841306. Etymology: named after the host genus, *Lithocarpus*. Diagnosis: morphologically distinct from the other *Monochaetia* species by hyaline conidia. Typus: China, Guangdong Province, Qingyuan City, Yangshan County, Guangdong Nanling Nature Reserve, on diseased leaves of Lithocarpus glaber, 4 December 2019, Shang Sun (holotype CAF 800018; ex-holotype culture CFCC 54509). Description: conidiomata in culture sporodochial, aggregated or solitary, erumpent, pulvinate, brown, 300 to 700 μm in diameter, exuding brown conidial masses. Conidiophores septate and branched, hyaline, thin walled. Conidiogenous cells hyaline, smooth, cylindrical to subcylindrical, annelidic, 5 to 22.5 by 2 to 3.5 μm, mean ± SD = 15.8 ± 4.5 by 2.9 ± 0.4 μm. Conidia fusoid, straight or slightly curved, 4-septate, smooth, constricted at the septa, without appendages (31 to) 31.5 to 34 (to 35) by (7.5 to) 8 to 8.5 (to 9) μm, mean ± SD = 33 ± 1.2 by 8.3 ± 0.4 μm (*n* = 50), L/W = 3.5 to 4.5; basal cell obconic with a truncate base, thin walled, hyaline, (5 to) 5.5 to 7.5 (to 8) μm long (without appendage); median cells 3, trapezoid or subcylindrical, pale brown, thick walled, the first median cell from the base (5.5 to) 6 to 7 (to 7.5) μm long, the second cell (6 to) 6.5 to 8.5 μm long, the third cell (6 to) 6.5 to 8.5 (to 9) μm long, altogether (18 to) 19 to 22.5 (to 23) μm long; apical cell conic with an acute apex, thin walled, hyaline, 5.5 to 6.5 μm long (without appendage); basal appendage single, unbranched, tubular, centric, straight or slightly bent, (6.5 to) 7 to 12.5 (to 13.5) μm long, mean ± SD = 9.7 ± 2.6 μm; apical appendage single, unbranched, tubular, centric, bent, (12 to) 13 to 23 (to 24) μm long, mean ± SD = 18 ± 4.8 μm. Sexual morph unknown. Culture characteristics: colonies on MEA flat, with ruffle sag on the surface and undulate edge, white to pale luteous, reaching 25 mm in diameter after 10 days at 25°C, forming brown conidiomata with brown conidial masses; colonies on PDA flat, spreading, with entire edge, white to pale luteous, reaching 25 mm in diameter after 10 days at 25°C, forming brown conidiomata with brown conidial masses. Additional material examined: China, Guangdong Province, Qingyuan City, Yangshan County, Guangdong Nanling Nature Reserve, on diseased leaves of Lithocarpus glaber, 4 December 2019, Shang Sun (culture CFCC 54448).

**FIG 6:**
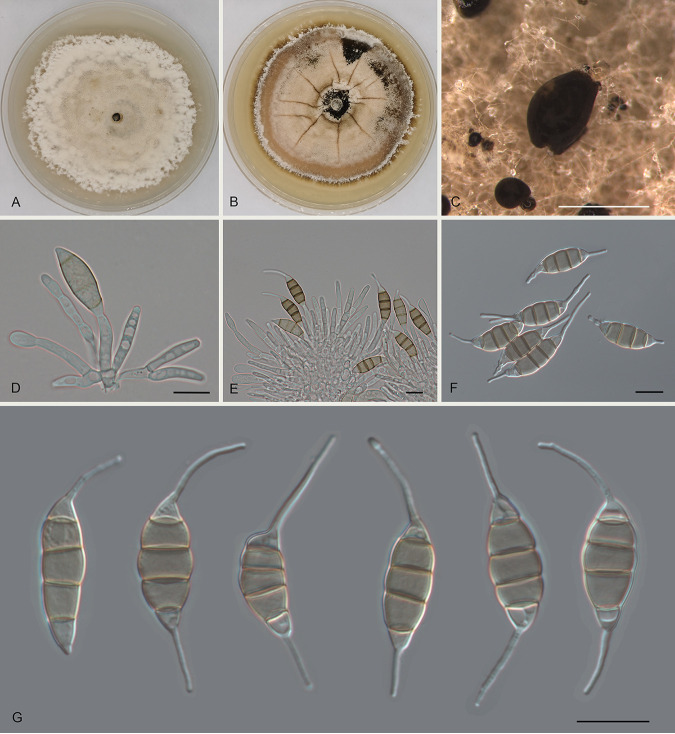
Morphology of Monochaetia quercicola (CFCC 55515). (A) Colony on PDA after 10 days at 25°C. (B) Colony on MEA after 10 days at 25°C. (C) Conidiomata formed on PDA. (D and E) Conidiogenous cells giving rise to conidia. (F and G) Conidia. Scale bars, 300 μm (C) and 10 μm (D to G).

Notes: Two isolates of Monochaetia quercicola, from leaf spots of Quercus acutissima and Q. aliena, formed a distinct clade in the phylogram close to M. ilicis and M. schimae (Fig. 1). However, Monochaetia quercicola can be distinguished from M. ilicis and M. schimae by wider conidia (7 to 9.5 μm versus 3 to 5 μm in M. ilicis versus 4.5 to 6 μm in M. schimae) (14, 16).

Monochaetia quercicola Ning Jiang sp. nov. (Fig. 6). MycoBank number MB846698. Etymology: named after the host genus, *Quercus*. Diagnosis: morphologically distinct from the phylogenetically related species of M. ilicis and M. schimae by wider conidia. Typus: China, Henan Province, Xinyang City, Shihe District, Jigong Mountain, on diseased leaves of Quercus aliena, 7 August 2019, Yong Li (holotype CAF 800019; ex-holotype culture CFCC 55515). Description: conidiomata in culture sporodochial, aggregated or solitary, erumpent, pulvinate, dark brown, 50 to 350 μm in diameter, exuding dark brown conidial masses. Conidiophores septate and branched, hyaline, thin walled. Conidiogenous cells hyaline, smooth, cylindrical to subcylindrical, annelidic, 8.5 to 21 by 2.5 to 3 μm, mean ± SD = 14.7 ± 3.6 by 2.8 ± 0.3 μm. Conidia fusoid, slightly curved, 4-septate, smooth, constricted at the septa, without appendages (19.5 to) 20.5 to 23 (to 25) by (7 to) 7.5 to 9 (to 9.5) μm, mean ± SD = 21.8 ± 1.4 by 8.2 ± 0.6 μm (*n* = 50), L/W = 2.3 to 3.3; basal cell obconic with a truncate base, thin walled, hyaline, 3 to 4.5 μm long (without appendage); median cells 3, trapezoid or subcylindrical, pale brown to brown, thick walled, the first median cell from the base (4 to) 4.5 to 6 μm long, the second cell 4.5 to 5 (to 5.5) μm long, the third cell (4 to) 4.5 to 5 (to 6) μm long, altogether (13 to) 13.5 to 15.5 μm long; apical cell conic with an acute apex, thin walled, hyaline, 3 to 4 (to 4.5) μm long (without appendage); basal appendage single, unbranched, tubular, centric, straight or slightly bent, (5 to) 6 to 11.5 μm long, mean ± SD = 8.7 ± 2.6 μm; apical appendage single, unbranched, tubular, centric, bent, (5 to) 9.5 to 17 (to 17.5) μm long, mean ± SD = 13.3 ± 3.8 μm. Sexual morph unknown. Culture characteristics: colonies on MEA flat, spreading, with radially folded surface and entire edge, sienna to hazel, reaching 60 mm in diameter after 10 days at 25°C, forming black conidiomata with black conidial masses; colonies on PDA flat with flocculent mycelium and undulate edge, white to luteous, reaching 60 mm in diameter after 10 days at 25°C, forming black conidiomata with black conidial masses. Additional material examined: China, Henan Province, Xinyang City, Shihe District, Jigong Mountain, on diseased leaves of Quercus acutissima, 7 August 2019, Yong Li (culture CFCC 55248).

**FIG 7:**
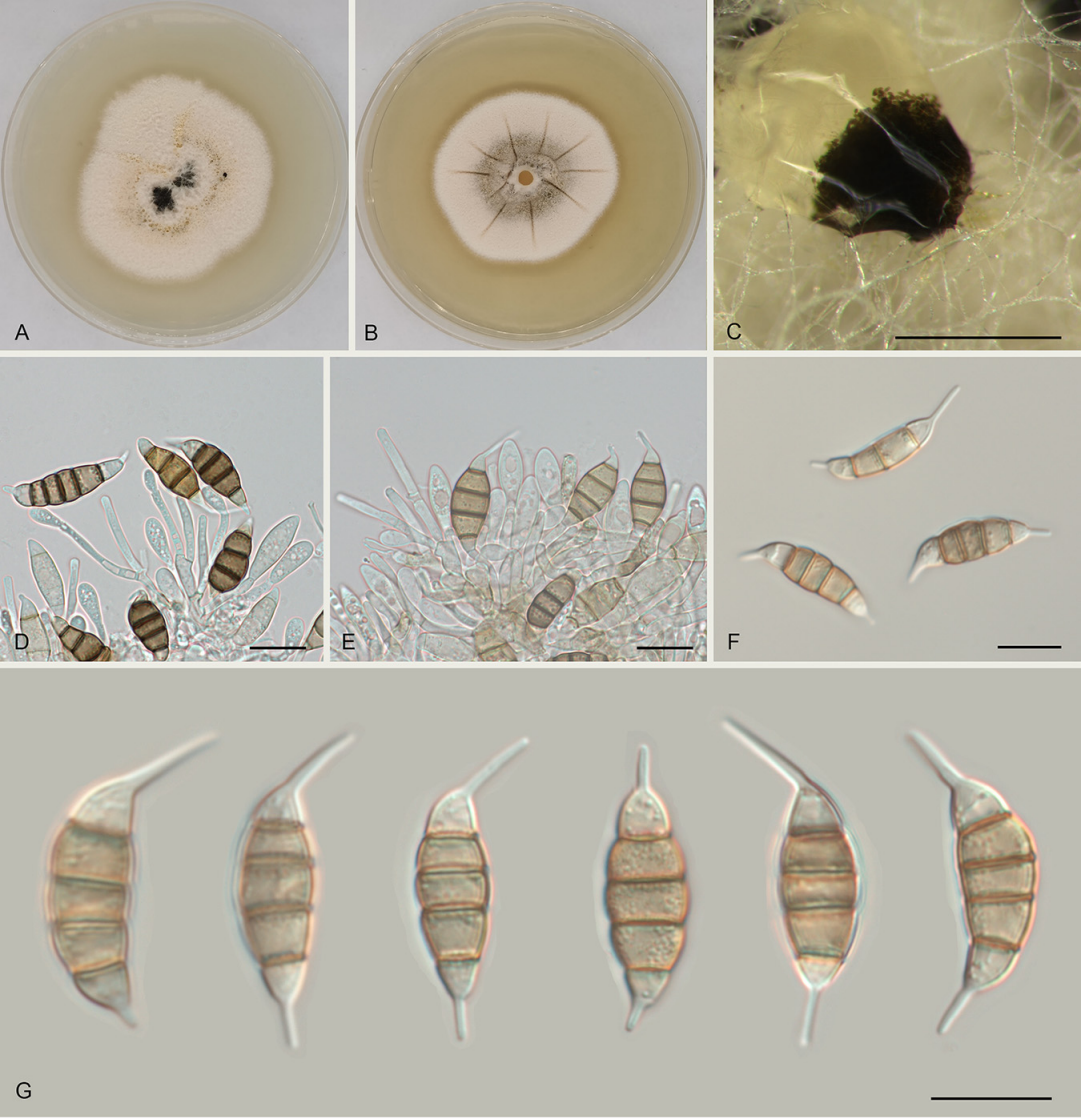
Morphology of Monochaetia shaanxiensis (CFCC 54419). (A) Colony on PDA after 10 days at 25°C. (B) Colony on MEA after 10 days at 25°C. (C) Conidioma formed on PDA. (D and E) Conidiogenous cells giving rise to conidia. (F and G) Conidia. Scale bars, 300 μm (C) and 10 μm (D to G).

Notes: Monochaetia shaanxiensis is phylogenetically close to M. hanzhongensis, M. kansensis, and M. lithocarpi (Fig. 1). However, M. shaanxiensis has a distinctly shorter apical appendage than M. lithocarpi (3.5 to 8 μm versus 13 to 23.5 μm in M. lithocarpi), a shorter basal appendage than M. kansensis (2 to 3 μm versus 3 to 15 μm in M. kansensis), and wider conidia than M. hanzhongensis (6.5 to 8.5 μm versus 5 to 6.5 μm in M. hanzhongensis) (Table 1).

Monochaetia shaanxiensis Ning Jiang sp. nov. (Fig. 7). MycoBank number MB841307. Etymology: named after the collection site of the type specimen, Shaanxi Province. Diagnosis: morphologically distinct from the phylogenetically related species of M. kansensis by shorter basal appendage. Typus: China, Shaanxi Province, Xian City, Zhouzhi County, Heihe Forest Park, on diseased leaves of Quercus baronii, 6 September 2019, Yong Li (holotype CAF 800020; ex-holotype culture CFCC 54419). Description: conidiomata in culture sporodochial, aggregated or solitary, erumpent, pulvinate, dark brown, 150 to 450 μm in diameter, exuding dark brown conidial masses. Conidiophores septate and branched, hyaline, thin walled. Conidiogenous cells hyaline, smooth, cylindrical to subcylindrical, annelidic, 11.5 to 20.5 by 2 to 3 μm, mean ± SD = 15.7 ± 2.7 by 2.4 ± 0.2 μm. Conidia fusoid, straight or slightly curved, 4-septate, smooth, slightly constricted at the septa, without appendages, (19 to) 19.5 to 21.5 (to 22) by 6.5 to 7.5 (to 8.5) μm, mean ± SD = 20.6 ± 0.9 by 7.1 ± 0.5 μm (*n* = 50), L/W = 2.5 to 3.2; basal cell obconic with a truncate base, thin walled, hyaline or pale brown, 3 to 4 (to 4.5) μm long (without appendage); median cells 3, trapezoid or subcylindrical, brown, thick walled, the first median cell from the base 4.5 to 5 (to 5.5) μm long, the second cell 3.5 to 4.5 μm long, the third cell (3 to) 3.5 to 4.5 μm long, altogether 11.5 to 13 μm long; apical cell conic with an acute apex, thin walled, hyaline, (4 to) 4.5 to 5.5 μm long (without appendage); basal appendage single, unbranched, tubular, centric, straight, 2 to 3 μm long, mean ± SD = 2.5 ± 0.3 μm; apical appendage single, unbranched, tubular, centric, straight, (3.5 to) 4 to 6.5 (to 8) μm long, mean ± SD = 5.1 ± 1.4 μm. Sexual morph unknown. Culture characteristics: Colonies on MEA flat, spreading, with radially folded surface and entire edge, luteous to olivaceous gray, reaching 35 mm in diameter after 10 days at 25°C, forming black conidiomata with black conidial masses; colonies on PDA flat, spreading, with flocculent aerial mycelium and undulate edge, white to pale luteous, reaching 45 mm in diameter after 10 days at 25°C, forming black conidiomata with black conidial masses.

## DISCUSSION

In the present study, 9 isolates from *Fagaceae* leaf spots in China were identified as 5 new species of *Monochaetia*. However, we investigated only 5 *Fagaceae* hosts of more than 320 reported species in China, indicating that many hidden *Monochaetia* species may remain to be discovered from *Fagaceae* in the future.

Species of *Monochaetia* are commonly known as leaf pathogens or saprobes and are sometimes isolated from air (4, 13 to 15). In previous studies, 8 species were recorded from fagaceous hosts, inhabiting leaves of *Castanea* or *Quercus* (4, 13, 15). Of these, 3 species (Monochaetia bicornis, Monochaetia concentrica, and Monochaetia hysteriiformis) have not yet been sequenced. In the present study, 5 additional new species were revealed from *Fagaceae* based on conidial characteristics and molecular phylogenetic evidence (Table 1). Two new species, Monochaetia lithocarpi and M. lithocarpicola, were discovered from *Lithocarpus*, which represents a new host genus for *Monochaetia*. However, no *Monochaetia* species are known from the residual 5 genera of *Fagaceae*; considering that leaf spot pathogens of these are still poorly studied in most areas, it is possible that additional *Monochaetia* species may be revealed from them.

As shown in Table 1, Monochaetia concentrica and M. kansensis can inhabit both *Castanea* and *Quercus* (15). Therefore, while host association may be indicative for some species, it cannot be generally considered a reliable characteristic to separate *Monochaetia* species. Most *Monochaetia* species sequenced are still known from only a few confirmed isolates from a few localities and, as with other genera of *Sporocadaceae*, the possibility that many species have wider host ranges than the few published records may suggest cannot be excluded.

Conidial morphology, including pigmentation, septation and wall ornamentation of wall cells, position of appendages with respect to the apical and basal cells, and the number and branching pattern of apical appendages, has been widely used to separate pestalotioid genera and species (10, 11, 13). In the present study, we discovered the first species having nearly hyaline to pale brown conidia in *Monochaetia*, M. lithocarpicola (Fig. 5). This suggests that pigmentation might not be a robust characteristic to distinguish genera in *Sporocadaceae*, which was similarly observed for *Allelochaeta* (20).

This study revealed five new *Monochaetia* species associated with *Fagaceae* leaf spot disease symptoms in China. Because Castanea mollissima is a widely cultivated fagaceous host in China (4), we tested the pathogenicity of M. castaneae (from Castanea mollissima), M. hanzhongensis (from Quercus variabilis), M. lithocarpi (from Lithocarpus glaber), M. lithocarpicola (from L. glaber), M. quercicola (from Quercus acutissima and Q. aliena) and M. shaanxiensis (from Quercus baronii) to healthy leaves of C. mollissima. Only M. castaneae successfully infected C. mollissima and caused brown lesions, which implied that the other five fungal species are not pathogenic to the important crop fagaceous species C. mollissima.

## MATERIALS AND METHODS

### Sample collection and isolation.

Investigations were conducted in Guangdong, Henan, and Shaanxi Provinces in China from 2016 to 2021 to collect diseased fagaceous leaf samples. A total of two host genera and five species in *Fagaceae*, namely, Lithocarpus glaber, Q. variabilis, Q. aliena, Q. acutissima, and Q. baronii, were investigated in the present study. The leaf samples were packed in paper bags and transferred to the laboratory for fungal isolation.

The diseased leaf samples were surface sterilized for 1 min in 75% ethanol, 3 min in 1.25% sodium hypochlorite, and 1 min in 75% ethanol, rinsed for 2 min in distilled water, and blotted on dry sterile filter paper. Then the diseased areas of the leaves were cut into pieces (0.5 by 0.5 cm) with an aseptic razor blade, transferred onto the surface of both PDA (200 g/L potato, 20 g/L dextrose, 20 g/L agar) and MEA (30 g/L malt extract, 5 g/L mycological peptone, 15 g/L agar) plates, and incubated at 25°C to obtain fungal hyphae. Hyphal tips were then removed to new PDA and MEA plates to obtain pure cultures. The cultures were deposited in the China Forestry Culture Collection Center (CFCC) (http://cfcc.caf.ac.cn/) and the specimens in the herbarium of the Chinese Academy of Forestry (CAF) (http://museum.caf.ac.cn).

### DNA extraction, sequencing, and phylogenetic analyses.

Genomic DNA was extracted from colonies grown on cellophane-covered PDA plates using a cetyltrimethylammonium bromide (CTAB) method (21). DNA quality was estimated by electrophoresis in 1% agarose gels, and the quality was measured with a NanoDrop 2000 spectrophotometer (Thermo Fisher Scientific, Waltham, MA, USA) following the user manual. The following primer pairs were used for amplification of the gene regions sequenced in the present study: ITS1/ITS4 for the 5.8S nuclear ribosomal DNA gene with the two flanking ITS regions (ITS1 and ITS2) (22), LR0R/LR5 for the nuclear ribosomal LSU region (23), EF1-728F/EF2 for the translation elongation factor 1-α (*tef1*) gene (24, 25), and Bt2a/Bt2b for the β-tubulin (*tub2*) gene (26). The PCR conditions were set as follows: an initial denaturation step of 5 min at 94°C, 35 cycles of 30 s at 94°C, 50 s at 52°C (ITS and LSU) or 54°C (*tef1* and *tub2*), and 1 min at 72°C, and a final elongation step of 7 min at 72°C. PCR amplification products were checked via electrophoresis in 2% agarose gels. DNA sequencing was performed using an ABI Prism 3730XL DNA Analyzer with a BigDye Terminator kit v.3.1 (Invitrogen, USA) at the Shanghai Invitrogen Biological Technology Company Ltd. (Beijing, China).

The quality of the amplified nucleotide sequences was checked and the sequences were assembled using SeqMan v.7.1.0. Reference sequences were retrieved from the National Center for Biotechnology Information (NCBI) database. Sequences were aligned using MAFFT v.7 (http://mafft.cbrc.jp/alignment/server) (27) and corrected manually using MEGA 6 (28). The nucleotide sequence data from the present study were deposited in GenBank, and the accession numbers are listed in Table 2. The phylogenetic analyses of the combined matrices were performed using ML and BI methods. ML analysis was implemented with the CIPRES Science Gateway portal (https://www.phylo.org) using RAxML-HPC BlackBox v.8.2.10 (29), employing a GTRGAMMA substitution model with 1,000 bootstrap replicates, while BI analysis was performed using a Markov chain Monte Carlo (MCMC) algorithm in MrBayes v.3.0 (30). Two MCMC chains were run, starting from random trees, for 1,000,000 generations, and trees were sampled every 100th generation, resulting in a total of 10,000 trees. The first 25% of trees were discarded as burn-in for each analysis. Branches with significant Bayesian posterior probabilities (BPPs) were estimated in the remaining 7,500 trees. Phylogenetic trees were viewed with FigTree v.1.3.1 and graphically processed with Adobe Illustrator CS5.

**TABLE 2:** Isolates and GenBank accession numbers used for phylogenetic analyses in this study

Species	Isolate	Host/substrate	Origin	GenBank accession no.			
				LSU	ITS	*tub2*	*tef1*
Monochaetia castaneae	CFCC 54354^*a*^	Castanea mollissima	China	MW166263	MW166222	MW218515	MW199741
M. castaneae	SM9-2	Castanea mollissima	China	MW166264	MW166223	MW218516	MW199742
M. dimorphospora	NBRC 9980	Castanea pubinervis	Japan	LC146750	LC146750	NA^*b*^	NA
M. hanzhongensis	CFCC 54451^*a*^^,^^*c*^	Quercus variabilis	China	OK339776	OK339747	OK358484	OK358475
M. ilicis	KUMCC 15-0520 a	*Ilex* sp.	China	KX984152	KX984153	MH061345	NA
Monochaetia junipericola	CBS 143391^*a*^	Juniperus communis	Germany	MH107947	MH107900	MH108045	MH108021
M. kansensis	PSHI2004 Endo1031	Cyclobalanopsis myrsinaefolia	China	DQ534036	DQ534045	DQ534048	NA
M. kansensis	PSHI2004 Endo1030	Cyclobalanopsis myrsinaefolia	China	DQ534035	DQ534044	DQ534047	NA
M. lithocarpi	CFCC 54402^*a*^^,^^*c*^	Lithocarpus glaber	China	OK339777	OK339748	OK358485	OK358476
M. lithocarpi	CFCC 54379^*c*^	Lithocarpus glaber	China	OK339778	OK339749	OK358486	OK358477
M. lithocarpi	CFCC 54500^*c*^	Lithocarpus glaber	China	OK339779	OK339750	OK358487	OK358478
M. lithocarpicola	CFCC 54509^*a*^^,^^*c*^	Lithocarpus glaber	China	OK339780	OK339751	OK358488	OK358479
M. lithocarpicola	CFCC 54448^*c*^	Lithocarpus glaber	China	OK339781	OK339752	OK358489	OK358480
M. massachusettsianum	CBS 146013^*a*^	Air	USA	MN567633	MN562126	NA	MN556824
M. monochaeta	CBS 115004	Quercus robur	Netherlands	MH554198	AY853243	MH554639	MH554398
M. monochaeta	CBS 199.82^*a*^	Quercus pubescens	Italy	MH554238	MH554018	MH554694	MH554452
M. monochaeta	CBS 315.54	*Quercus* sp.	UK	MH554249	MH554030	NA	MH554465
M. monochaeta	CBS 546.80	Culture contaminant	Netherlands	MH554270	MH554056	MH554732	MH554491
M. monochaeta	CBS 658.95	Quercus robur	Netherlands	MH554276	MH554063	NA	MH554499
M. quercicola	CFCC 55515^*a*^^,^^*c*^	Quercus aliena	China	OK339782	OK339753	OK358490	OK358481
M. quercicola	CFCC 55248^*c*^	Quercus acutissima	China	OK339783	OK339754	OK358491	OK358482
M. quercus	CBS 144034^*a*^	Quercus eduardi	Mexico	MH554365	MH554171	MH554844	MH554606
M. schimae	SAUCC212201^*a*^	Schima superba	China	NA	MZ577565	OK104867	OK104874
M. schimae	SAUCC212202	Schima superba	China	NA	MZ577566	OK104868	OK104875
M. shaanxiensis	CFCC 54419^*a*^^,^^*c*^	Quercus baronii	China	OK339784	OK339755	OK358492	OK358483
M. sinensis	HKAS10065^*a*^	*Quercus* sp.	China	MH115994	MH115995	MH115999	NA
Monochaetia sp.	CBS 101009	Air	Japan	MH554176	MH553953	MH554612	MH554371
Neopestalotiopsis cubana	CBS 600.96^*a*^	Leaf litter	Cuba	KM116253	KM199347	KM199438	KM199521
Pestalotiopsis australasiae	CBS 114126^*a*^	*Knightia* sp.	New Zealand	NA	KM199297	KM199409	KM199499

a Ex-type strain.

b NA, not available.

c Isolate generated in the present study.

For closely related species with similar morphology, ITS, LSU, *tef1*, and *tub2* sequences of the species were pairwise compared. For this, the sequences of species pairs were aligned, and the parts containing leading/trailing gaps were removed. Sequence differences of this alignment are recorded in the following way: number of nucleotide substitutions (excluding insertions and gaps)/total number of nucleotide characters, percentage of sequence substitutions, and numbers of insertions and gaps.

### Morphology.

The morphological data for the isolates collected in the present study were obtained from sporulating pure cultures grown on PDA or MEA plates in the dark at 25°C. The conidiomata were observed and photographed using a dissecting microscope (M205 C; Leica, Wetzlar, Germany). Microscope slides of conidiogenous cells and conidia were prepared in tap water, and the slides were examined and photographed with an Axio Imager 2 microscope (Zeiss, Oberkochen, Germany) equipped with an Axiocam 506 color camera or an Eclipse 80i microscope (Nikon, Tokyo, Japan) equipped with a Nikon digital sight DS-Ri2 camera, using differential interference contrast (DIC) illumination. For measurements, 50 conidia were randomly selected. Measurements of the conidia are reported as maximum and minimum (in parentheses) and the range representing the mean ± SD of the number of measurements indicated. Culture characteristics were recorded from 9-cm PDA or MEA plates after 10 days of incubation at 25°C in the dark. To enable comparison of species growing on fagaceous hosts, measurement data and sequence data available are summarized in Table 1.

### Pathogenicity.

The isolates representing Monochaetia castaneae (CFCC 54354), M. hanzhongensis (CFCC 54451), M. lithocarpi (CFCC 54402), M. lithocarpicola (CFCC 54509), M. quercicola (CFCC 55515), and M. shaanxiensis (CFCC 54419) were selected for inoculations. Healthy leaves of Castanea mollissima were washed in distilled water, surface sterilized in 75% ethanol for 1 min, rinsed in distilled water, and then surface wounded with a sterile needle in both the left and right portions of the leaves. Conidia of the six *Monochaetia* species were harvested from 4-week-old PDA plates with 10 mL of sterilized water, and the conidial suspension was filtered through two layers of cheesecloth to eliminate debris and mycelia. The conidial suspension was adjusted to a final inoculum concentration of 1 × 10^6^ conidia/mL with sterile deionized water. Then, 10 μL of conidial suspension was placed in the left portion of the leaves; sterile water inoculated in the right portion served as the negative control. Each treatment had five replicates, and the experiment was carried out twice. The inoculated leaves were placed in transparent plastic bags at 25°C and >90% humidity in the dark for 10 days. After the appearance of symptoms, fungal isolates were reisolated from the infected leaves and identified based on the morphological and phylogenetic analyses to fulfill Koch’s postulates.
